# Poor handling of continuous predictors in clinical prediction models using logistic regression: a systematic review

**DOI:** 10.1016/j.jclinepi.2023.07.017

**Published:** 2023-09

**Authors:** Jie Ma, Paula Dhiman, Cathy Qi, Garrett Bullock, Maarten van Smeden, Richard D. Riley, Gary S. Collins

**Affiliations:** a Centre for Statistics in Medicine, Nuffield Department of Orthopaedics, Rheumatology and Musculoskeletal Sciences, University of Oxford, Oxford OX3 7LD, United Kingdom; b Population Data Science, Swansea University Medical School, Faculty of Medicine, Health and Life Science, Swansea University, Singleton Park Swansea, SA2 8PP, Swansea, United Kingdom; c Department of Orthopaedic Surgery, Wake Forest School of Medicine, Winston-Salem, NC, USA; d Centre for Sport, Exercise and Osteoarthritis Research Versus Arthritis, University of Oxford, Oxford, United Kingdom; e Julius Center for Health Sciences and Primary Care, University Medical Center Utrecht, Utrecht University, Utrecht, The Netherlands; f Institute of Applied Health Research, College of Medical and Dental Sciences, University of Birmingham, Birmingham B15 2TT, United Kingdom

**Keywords:** Prediction, Continuous predictors, Clinical prediction model, Model development, Nonlinear methods, Statistical modelling

## Abstract

**Background and Objectives:**

When developing a clinical prediction model, assuming a linear relationship between the continuous predictors and outcome is not recommended. Incorrect specification of the functional form of continuous predictors could reduce predictive accuracy. We examine how continuous predictors are handled in studies developing a clinical prediction model.

**Methods:**

We searched PubMed for clinical prediction model studies developing a logistic regression model for a binary outcome, published between July 01, 2020, and July 30, 2020.

**Results:**

In total, 118 studies were included in the review (18 studies (15%) assessed the linearity assumption or used methods to handle nonlinearity, and 100 studies (85%) did not). Transformation and splines were commonly used to handle nonlinearity, used in 7 (*n* = 7/18, 39%) and 6 (*n* = 6/18, 33%) studies, respectively. Categorization was most often used method to handle continuous predictors (*n* = 67/118, 56.8%) where most studies used dichotomization (*n* = 40/67, 60%). Only ten models included nonlinear terms in the final model (*n* = 10/18, 56%).

**Conclusion:**

Though widely recommended not to categorize continuous predictors or assume a linear relationship between outcome and continuous predictors, most studies categorize continuous predictors, few studies assess the linearity assumption, and even fewer use methodology to account for nonlinearity. Methodological guidance is provided to guide researchers on how to handle continuous predictors when developing a clinical prediction model.

**What is new?**

**Key findings**
• Very few studies assess the linearity assumption or report methods to assess the functional form of continuous predictors.
• Studies continue to categorize and dichotomize continuous predictors leading to potential loss of predictive accuracy.

**What this adds to what is known?**
• We add to the building body of literature showing that continuous predictors are poorly handled in prediction model research.
• Methodological guidance is provided to guide researchers on how to handle continuous predictors when developing a clinical prediction model.

**What is the implication and what should change now?**
• We encourage researchers to consider methods to handle continuous predictors during study design and protocol development, prior to any analysis, which should then be reported clearly and transparently in the final report.

## 1 Introduction

Prediction models are used to calculate an individual's predicted value or estimated risk of a health outcome [[1], [2], [3], [4]]. They guide clinical decision-making by informing diagnosis (probability of having a disease) and prognosis (probability of future health outcomes).

When developing a clinical prediction model, multiple predictors are considered ranging from patient characteristics, blood test results, data from images, and patient-reported measures. These predictors are typically combined into an equation using a regression model, though machine learning approaches (e.g., random forests, deep learning) are increasingly being used. Predictors considered for inclusion in the prediction model using regression will often include a continuous predictor (e.g., age, systolic blood pressure, body mass index). How continuous predictors are handled during model development will influence model predictions for an individual and thus can have an impact on subsequent clinical decisions and patient care. It is therefore important that researchers carefully consider how continuous predictors are examined and included during the analysis to ensure a robust model is developed and provides the most accurate predictions.

Potential approaches for handling continuous predictors are to (1) include them as a linear term, indicating that the functional relationship between outcome and the continuous predictor is assumed to be linear; (2) categorize them into two or more groups; and (3) use transformations, splines, or fractional polynomials to select their functional (potentially nonlinear relationship between the outcome and continuous predictors) form of continuous predictors. Including a continuous predictor as a linear term assumes that one-unit increase in the predictor across all values of the predictor has the same effect on the outcome. Failure to model the functional form appropriately (i.e., the shape of the relationship between a continuous predictor and the outcome) can lead to a substantial loss of statistical power to detect and model the true underlying relationship. In turn, this may produce a prediction model with worse predictive performance and inaccurate predictions to base clinical decisions on, which can adversely influence patient care [[5], [6], [7], [8], [9], [10]]. Categorizing continuous predictors into two or more groups, a practice that is widely discredited, may lead to a prediction model with weaker performance compared to a model where the functional form has been modeled appropriately [7]. Furthermore, as noted by Collins et al., “categorizing continuous predictors leads to poor models, as it forces an unrealistic, biologically implausible, and ultimately incorrect (step) relationship onto the predictor and discards information” [7,11].

How continuous predictors will be handled and analyzed (including assessment of modeling assumptions) should ideally be considered at the design stage when developing the study protocol (or statistical analysis plan). Furthermore, how continuous predictors are to be handled during model development should be accounted in the sample size calculation [[12], [13], [14]]. Once sufficient data are obtained, the functional form of continuous predictors should ideally be analyzed using recommended techniques such as predictor transformation, restricted cubic splines, and fractional polynomials [7,[15], [16], [17], [18], [19]].

Though research recommendations have long been established for handling continuous predictors [[8], [9], [10],^15^,^16^], it is unclear how frequently continuous predictors are actually examined, included, and reported when developing a clinical prediction model. Existing reviews of evaluating the methodological conduct of prediction model studies have observed that continuous predictors are frequently handled poorly (e.g., categorized [[20], [21], [22]]) but do not go into more detail. The aim of this article is to delve deeper and review how continuous predictors were included in studies developing a clinical prediction model in low-dimensional settings. To do this, we sought to examine study quality and whether authors considered the functional form of continuous predictors in common prediction modeling scenarios (e.g., predicting binary outcome using logistic regression), including the reporting of checking linearity or if nonlinearity was considered and how.

## 2 Methods

We conducted a systematic review of studies developing a diagnostic, prognostic, or risk prediction model for a binary outcome that examined at least one continuous candidate predictor and used logistic regression. The study protocol is available on the Open Science Framework (DOI: osf.io/TMHU9) [23]. We reported our study following the Preferred Reporting Items for Systematic Reviews and Meta-Analyses (PRISMA) guidelines [24].

### 2.1 Information sources

A systematic search was carried out using an electronic medical literature database (PubMed) on August 03, 2020, for published studies developing a clinical prediction model in any clinical specialty. We searched for studies published between July 01, 2020, and July 30, 2020.

The search strategy was formed by combining prediction, modeling, and model performance search terms. Prediction search terms included “prediction,” “prognostic,” and “diagnostic.” Modeling search terms included “model,” “logistic,” and “regression.” Model performance search terms included “discrimination,” “calibration,” and “area under the curve.” Publications satisfying the prediction, modeling, and model performance search strings were then restricted to studies published within the search dates. The complete search strategy is provided in Supplementary Box 1.

### 2.2 Eligibility criteria

Articles were included if they met the following inclusion criteria:

• Studies developing a clinical prediction model (diagnosis or prognosis):
  • for any clinical specialty
  • for binary outcomes
  • using logistic regression (including penalization approaches, for example, least absolute shrinkage and selection operator [LASSO] regression)
  • including at least one continuous candidate predictor (e.g., a continuous measurement, such as, age, height, and hemoglobin value)
  • using any study design:
    ◦ experimental studies (e.g., randomized trials)
    ◦ observational studies (e.g., cohort studies, case–control studies, registry-based studies)
• English language studies
• Primary research studies

Articles were excluded using the following criteria:

• Studies developing a prediction model
  • using artificial intelligence or machine learning
  • using images or information extracted from images (imaging studies)
  • using genetic or omics data (genetic studies)
  • using molecular data (molecular studies)
  • using lab-based or animal data (lab-based studies)
• Risk or prognostic factor studies, primarily interested in the association of individual risk or prognostic factors with a particular outcome
• Reviews of clinical prediction models
• Studies only evaluating the performance of a clinical prediction model (i.e., validation studies)
• Conference abstracts
• Studies with unavailable full text

Studies that developed machine learning models (e.g., random forests, support vector machines) and compared them to statistical regression-based models were also included; however, only information on the logistic regression model was extracted. When an article reported more than one regression model, we extracted information on only the first model that was mentioned.

### 2.3 Study selection, data extraction, and management

Studies published in July 2020 were selected to provide a snapshot sample of studies. Publications retrieved from PubMed were imported into Endnote reference software [25] where duplications were removed. Publications were then imported into Rayyan [26] web application where they were again checked for duplicates (and duplicates removed) and screened for the title and abstract of articles against the eligibility criteria and after which screened for full-text inclusion.

Two researchers (J.M., P.D.) independently screened the titles and abstracts of the identified publications and reviewed the full text of eligible publications. Two researchers, from a combination of four reviewers (J.M., P.D., G.B., and C.Q.), independently extracted data from eligible publications. Disagreements were discussed and adjudicated by a fifth reviewer (G.S.C.), where necessary.

The data extraction form was developed based on the Transparent Reporting of a multivariable prediction model for Individual Prognosis or Diagnosis (TRIPOD) guideline [27] and the CHecklist for critical Appraisal and data extraction for systematic Reviews of prediction Modeling Studies [28]. These checklists were used to guide the data extraction of methods for handling continuous predictors in the analysis and methods to assess and explore the functional form and reporting of continuous predictors included in the final model. The data extraction form was piloted on five studies, inconsistency or difficulties were discussed, and the extraction form was amended accordingly. The data extraction form was implemented in Microsoft Excel.

### 2.4 Data items

Descriptive information was extracted on the overall publication, including items on study design, source of data, target population, outcome of prediction, and the type of model used. Extraction of methodological items included the number of candidate predictors, number of continuous predictors, indication (and details) of whether the linearity assumption was examined, details of any categorisation (including how cut-points were determined), common methods to handle nonlinear predictors (described in Box 1), details of the methods used to handle nonlinear predictors (e.g., knot location for restricted cubic splines, polynomial order for fractional polynomials), and frequency and details of nonlinear terms included in the final model. The number of candidate predictors and the number of candidate predictor parameters were considered “reported” if a total number was provided in the article or if the number could be counted. Other information extracted included the sample size and number of events, model discrimination, and calibration. For discrimination, we extracted the c-statistic, and for calibration, we extracted information on the presence of a calibration plot and any estimates of the calibration slope and intercept.

### 2.5 Data analysis

Data were summarized using descriptive statistics and a narrative synthesis. Results are presented overall for studies that explored the functional form of continuous predictors (i.e., checked the linearity assumption or used methods such as transformation or restricted cubic splines) and studies that did not explicitly consider the functional form (i.e., no mention of checking linearity and did not mention using any transformations). The number of candidate predictors and sample size used in studies were described using median, interquartile ranges (IQRs), and ranges. We also calculated the proportion of candidate predictors that were continuous and that were included in the final developed model. We calculated the number of events per predictor parameter by dividing the number of events used to develop the model by the number of candidate predictor parameters (number of degrees of freedom associated with the candidate predictions, calculated by the study team). Results for discrimination and calibration were summarized for each model. Data were exported to and analyzed in R [29]. We will derive a 95% confidence interval (CI) to quantify the uncertainty from our sample to make inference to a wider standard population of binary logistic regression model studies based on the findings from our sample by using “exactci” [30] package with Clopper–Pearson method in R.

## 3 Results

**Fig. 1:**
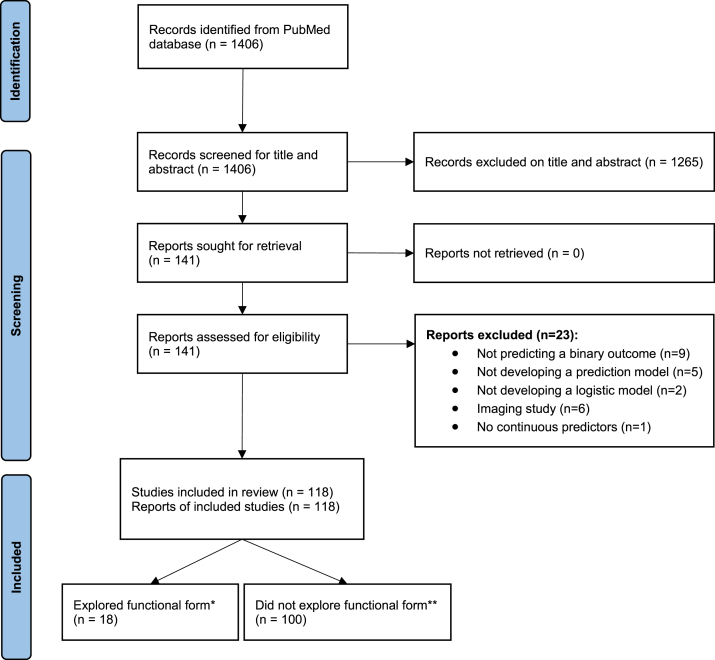
Preferred Reporting Items for Systematic Reviews and Meta-Analyses (PRISMA) flowchart. ∗ Reported checking the linearity assumption or reported methods to handle nonlinearity, such as transformation or restricted cubic splines. ∗∗ Did not report checking the linearity assumption and did not report using methods to handle nonlinearity. (For interpretation of the references to color in this figure legend, the reader is referred to the Web version of this article).

The search string identified a total of 1,406 publications indexed on PubMed between July 01, 2020, and July 30, 2020. Title and abstract screening excluded 1,265 publications, and full-text screening excluded a further 23 articles that did not meet inclusion criteria (e.g., were not predicting binary outcomes, did not develop a logistic prediction model, used images or data from images to predict the outcome, and did not include continuous candidate predictors). In total, 118 studies met eligibility criteria, including at least one continuous predictor, and were included in this review. The PRISMA flowchart is provided in Fig. 1, and a full reference list of included studies is provided in Supplementary Table 1.

### 3.1 Study design characteristics

Studies were mainly developed using an existing dataset (*n* = 71/118, 60.2%, CI: 50.9%–68.7%). Eighty-eight studies (*n* = 88/118, 74.6%, CI: 65.7%–81.9%) were prognostic studies, and a complication (e.g., bleeding, infection) was the most prevalent outcome being predicted (*n* = 28/118, 23.7%, CI: 16.9%–32.2%). Of the 118 studies developing a logistic regression model, 12 studies applied penalization methods (e.g., LASSO, elastic net) (10.2%, CI: 5.8%–17.3%).

Eighteen studies (*n* = 18/118, 15.3%, CI: 9.6%–22.8%) explored the functional form of their continuous predictors either by reporting that linearity was checked or by describing methods to handle nonlinearity. One hundred studies (*n* = 100/118, 84.7%, CI: 77.2%–90.4%) did not report exploring the functional form of their continuous predictors (i.e., checking linearity of their continuous predictors or describing any methods to handle nonlinearity). Study characteristics of included studies are presented in Table 1.

**Table 1:** Study characteristics

Study characteristic	All studies (*n* = 118)
	*n* (%)
Study type	
Development only	99 (83.9)
Development with external validation	19 (16.1)
Study design/data source	
Prospective cohort	24 (20.3)
Existing cohort data	71 (60.2)
Routinely collected data	13 (11.0)
Nested case–control	5 (4.2)
Existing data	4 (3.4)
Randomized trial	1 (0.8)
Outcome type	
Prognosis	88 (74.6)
Diagnosis	30 (25.4)
Explored functional form	18 (15.3)
Functional form assessed^a^	*14 (11.9)*
Checked linearity assumption and shows linearity	*2 (1.7)*
Checked linearity assumption and categorized	*2 (1.7)*
Did not explore functional form^b^	100 (84.7)
Implicitly assumed linearity for all continuous predictors	*38 (32.2)*
Categorized all continuous predictors	*40 (33.9)*
Both categorized and assumed linearity	*22 (18.6)*

The denominator for the italic values after ‘Explored functional form’ is 18 (e.g., 14/18 (11.9%) assessed functional form, 2/18 (1.7%) Checked linearity assumption and shows linearity, and 2/18 (1.7%) Checked linearity assumption and categorized. The denominator for the italic values after ‘Did not explore functional form’ is 100 (e.g., 38/100 (32.2%) Implicitly assumed linearity for all continuous predictors, 40/100 (33.9%) Categorized all continuous predictors, and 22/100 (18.6%) Both categorized and assumed linearity.

a Reported checking the linearity assumption or reported methods to handle nonlinearity, such as transformation or restricted cubic splines.

b Did not report checking the linearity assumption and did not report using methods to handle nonlinearity.

### 3.2 Predictors and sample size

All candidate predictors were clearly reported for nearly all models, either presented as a total number or were able to be counted (*n* = 115/118, 97.5%, CI: 92.6%–99.3%). A median of 19 candidate predictors were considered per model (range: 5-838) (Table 2). A median of six candidate continuous predictors were considered per model (range: 1-30), with a median of six predictors included in the final model (range: 1-163).

**Table 2:** Summary description of candidate predictors, degrees of freedom (candidate predictor parameters), continuous predictors, sample size, and events per predictor parameter used for model development

Prediction model information	Reported in the study^a^	Value
	*n* (%)	Median (Q1, Q3)
Candidate predictors clearly reported	115 (97.5)	19 (13, 28)
Degrees of freedom clearly reported	110 (93.2)	23 (16, 31)
Total continuous candidate predictors	109 (92.4)	6 (4, 11)
Final model predictors reported	110 (93.2)	6 (4, 8)
Total available sample size reported^b^	117 (99.2)	666 (283, 3,013)
Total available events reported^b^	110 (93.2)	137 (55, 406)
Sample size actually used to develop the model^b^	118 (100.0)	573 (222, 2,179)
Number of events actually used to develop the model^b^	95 (80.5)	100 (45, 237)
No. of events per predictor parameter used to develop the model (calculated)^c^	83 (67.5)	4 (2.2, 11.6)

Q1, lower quartile; Q3, upper quartile.

a The number of studies where this information was reported out of a total of 118 studies.

b Total available sample size and the number of events refer to the total amount of data available to be used to develop the model before any potential discarding of data or data splitting into development (“train") and internal validation (“test”) datasets. The sample size actually used and the number of events refer to the actual size of the data that was used to develop the models after any discarding of data or data splitting.

c Estimated using information reported in the primary studies.

Nine studies reported a sample size calculation to develop their models (*n* = 9/118, 7.6%, CI: 3.9%–13.9%), none of which accounted for the potential inclusion of nonlinear terms or additional parameters which would need to be estimated for categorical predictors with three or more categories. A median of 666 (range: 37-345718) individuals and 137 (range: 8-48262) events were used for model development.

Combining the number of candidate predictor parameters with the number of events resulted in a median of four events available per predictor (IQR: 2.2 to 11.6, *n* = 83 models). A higher number of events per predictor parameter was used in studies that explored the functional form of their continuous predictors (median: 10.4; IQR: 7 to 45.2; range: 0.8-1,141.5, *n* = 18 models) compared to studies that did not (median: 3.6; IQR: 1.6 to 12.6; range: 0.3-1,987.3, *n* = 100 models).

### 3.3 Handling continuous predictors

#### 3.3.1 Checking the linearity assumption

Of the 100 studies that did not report assessing the linearity assumption or report methods to handle nonlinearity, all continuous predictors were implicitly assumed and treated as linear for 38 studies (*n* = 38/100, 38%, CI: 28.9%–48.0%), and for each of the remaining 62 studies (*n* = 62/100, 62%, CI: 52.0%–71.1%), at least one continuous predictor was categorized.

Of the 18 studies for which the linearity assumption was checked or methods to handle nonlinearity were reported, 16 studies explicitly reported checking the linearity assumption (*n* = 16/18, 88.9%, CI: 67.0%–98.0%), and for two studies, it was unclear (i.e., nonlinear terms were reported in the analysis, e.g., squared and cube-root functions, without explicit assessment of linearity) [33,34]. Four studies presented a plot of continuous predictors to demonstrate the shape of the relationship between the continuous predictors and the outcome (*n* = 4/18, 22.2%, CI: 8.0%–47.1%): one study used a locally weighted scatterplot smoothing [35], one study used splines [36], one study plotted the continuous predictor against the log-odd of the outcome [37], and for one study, the categorized age was plotted against the outcome [38]. The linearity assumption was commonly assessed at the univariable analysis level (*n* = 10/18, 55.6%, CI: 33.0%–76.4%) but was unclear for seven studies (*n* = 7/18, 38.9%, CI: 18.5%–62.5%). One study assessed the linearity assumption after adjustment for other predictors.

#### 3.3.2 Categorization

Categorization of at least one continuous predictor was carried out in 67 studies (*n* = 67/118, 56.8%, CI: 47.5%–65.7%), including 62 studies that did not explore functional form of their continuous predictors (*n* = 62/67, 92.5%, CI: 83.7%–97.0%) and five studies that did explore functional form (*n* = 5/67, 7.5%, CI: 3.0%–16.3%). Of these five studies, two assessed the linearity assumption but still categorized all their continuous predictors [38,39], and three studies categorized their continuous predictors in addition to nonlinear terms (that accounted for their functional form) [33,40,41]. Forty-two studies (62.7%, CI: 50.3%–73.9%) categorized all their continuous predictors. Dichotomization of continuous predictors was the most prevalent approach (*n* = 40/67, 59.7%, CI: 47.4%–71.0%), while the remaining studies used three categories (*n* = 6), four categories (*n* = 3), five categories (*n* = 1), six categories (*n* = 2), and a mixed number of categories (*n* = 15).

Most studies provided no rationale when categorizing their continuous predictors (*n* = 56/67, 83.6%, CI: 72.5%–91.0%). One study determined their categorization cutoff values by assessing sensitivity of univariable models (“we analyzed the area under the curve to determine the best cutoff point of the parameters in the derivation cohort”) [42], one study dichotomized using the mean [43], two studies used quantiles [41,44], and one study used the outcome prevalence [45]. Four studies reported using clinically informed cutoffs [[46], [47], [48], [49]], and two studies claimed using cutoffs informed by previous literature [50,51].

#### 3.3.3 Exploring functional form

Of the 18 studies exploring functional form, six studies used (restricted) cubic splines to assess and capture the functional form of their continuous predictors (*n* = 6/18, 33.3%, CI: 15.6%–58.6%). Of these, one study concluded that the relationship between the continuous predictor and the outcome was linear (“the relationship between Common Procedural Terminology-specific complication event rate and the probability of any complication was approximately linear when visually inspected using a cubic smoothing spline.” [36]) and so did not include any nonlinear terms in their final model, and one study did include a nonlinear term in their final model without providing any additional information to support the inclusion of nonlinear terms [52]. Three models reported the number of knots in their cubic splines analysis [35,53,54], and three models reported the knot location [35,52,54].

Fractional polynomials were used in three studies (*n* = 3/18, 11.1%, CI: 4.7%–41.4%). One study used fractional polynomials to examine the linearity assumption and concluded that the continuous predictors could be assumed to be linear [55]. One study reported the order of the fractional polynomials [56], and for one study, the fractional polynomial order was unclear, and nonlinear terms were not included in the final model stating in the methods that “polynomial relationships for continuous covariates were also explored” and in the discussion that “complex nonlinear relationships between the covariates and the outcome that are difficult to explicitly capture even with the use of techniques such as including polynomial terms or cubic spline” [41].

Seven studies applied a transformation to their continuous predictors (*n* = 7/18, 38.9%, CI: 18.5%–62.5%): three studies used a log (base 10) transformation [40,57,58], two studies used quadratic transformations [59,60], one study used a squared transformation [33], and one study used a cube-root transformation [34].

Twelve studies developed their model using penalized regression (ten used LASSO regression, one used Ridge regression, and one used elastic net), and only two of these studies explored the functional form of the continuous predictors by using transformation and cubic spline [36,60].

### 3.4 Model presentation

Eighty-one models (*n* = 81/118, 68.4%, CI: 59.8%–76.5%) were inadequately presented, precluding them to be used or applied on a new individual as they did not report all the necessary information: 22 models did not report any regression coefficients or the intercept, and 59 models reported the regression coefficients but not the intercept. Only 37 models were fully reported and provided the necessary model regression coefficients with the intercept (*n* = 37/118, 31.4%, CI: 23.5%–40.2%).

Ten models (*n* = 10/18, 55.6%, CI: 33.0%–76.4%) out of those that used nonlinear terms included the nonlinear terms in the final model, of which eight models (*n* = 8/10, 80%, CI: 44.7%–96.3%) reported the intercept and all parameter estimates of the model. Seven models did not include nonlinear terms in their final model, and for one model, it was unclear. Of the eight models including nonlinear terms in the final model, two reported the restricted cubic spline terms (however, only one reported the required spline cut points and parameter estimates, while the second study did not report the spline cut points), two reported the fractional polynomial terms, and four reported the transformation term. Further details on the reporting and presentation of the nonlinear terms are provided in Supplementary Table 2.

### 3.5 Validation and model performance

Bootstrapping was the most common method to internally validate models where functional form had been explored (*n* = 8/18, 44.4%, CI: 23.6%–67.0%), and the split sample approach was most common in studies that did not explore functional from of their continuous predictors (*n* = 40/100, 40%, CI: 30.6%–50.0%).

Discrimination measures, such as the area under the receiver operating characteristic curve and analogous measures (e.g., c-statistic), were reported in almost all studies (*n* = 116/118, 98%, CI: 93.8%–99.7%), with similar levels of reporting between studies that explored functional form and studies that did not. However, calibration was more poorly reported in comparison to discrimination measures with about half of studies reporting recommended calibration metrics (i.e., including a calibration plot or reporting the calibration slope or intercept) (*n* = 63/118, 53.4%, CI: 44.1%–62.3%). Reporting the recommended calibration metrics was higher in studies that explored the functional form of their continuous predictors (*n* = 13/18, 72.2%, CI: 47.1%–88.4%) compared to studies that did not (*n* = 50/100, 50%, 40.0%–60.0%). However, comparable levels of studies reported the (unrecommended and uninformative) Hosmer–Lemeshow test as a measure of calibration (explored functional form: 3/18, 17% [4.7%–41.4%] vs. did not explore functional form: 18/100, 18% [11.3%–26.9%]). Additional information on the approaches used to internally validate the models and the reporting and summary of model performance measures are presented in Supplementary Tables 3 and 4 and Figure 1.

### 3.6 Reporting standards

Nineteen studies mentioned using a reporting guideline (*n* = 19/118, 16.1%, CI: 10.4%–24.1%), of which 16 studies used the TRIPOD reporting guideline. One study used STROCSS 2021: Strengthening the reporting of cohort, cross-sectional and case–control studies in surgery, one study used the Strengthening the Reporting of Observational Studies in Epidemiology (STROBE) Statement, and one study used the Consolidated Standards of Reporting Trials 2010 Statement: updated guidelines for reporting parallel group randomized trials. A higher proportion of studies addressing nonlinear continuous predictors used the recommended TRIPOD reporting guideline (*n* = 7/18, 38.9%, CI: 18.5%–62.5%) compared to studies that did not (9/100, 9%, CI: 4.6%–16.4%).

## 4 Discussion

### 4.1 Summary of findings

We reviewed 118 studies describing the development of a clinical prediction model for a binary outcome using logistic regression that included at least one continuous candidate predictor. Very few studies assessed the linearity assumption or reported methods to assess the functional form of their continuous predictors using simple transformations, restricted cubic splines, or fractional polynomials. For one study, it was also unclear if fractional polynomials (or other polynomials forms) were indeed used as the details were unclear, and nonlinear terms were not included in the final model [38]. We included this study in the “exploring functional form” group as they reported in the methods that “polynomial relationships for continuous covariates were also explored” and reported in the discussion that “complex nonlinear relationships between the covariates and the outcome that are difficult to explicitly capture even with the use of techniques such as including polynomial terms or cubic spline.” So, even for studies exploring the functional form of their continuous predictors, there remains ambiguity around what was done and poor reporting.

Studies more often assumed the relationship between continuous predictors and the outcome to be predicted was linear, with no attempt to explore whether this assumption was true. Many studies implicitly assumed linearity (i.e., they did not report checking this assumption), possibly unaware that simply “including” a predictor in the model assumes that the predictor is linearly associated with the outcome that needs to be checked or appropriately modeling to ensure the validity of the developed model (for logistic regression, linearity on the logit scale). Other studies categorized continuous predictors, which will ultimately lead to models with a loss in accuracy, leading to potentially harmful outcomes if decisions were to be made using these predictions. The very few studies that assessed the linearity assumption or reported methods to assess the functional form of continuous predictors used simple transformations, restricted cubic splines, or fractional polynomials. However, even in some of these studies, categorization of all or some predictors was carried out.

We found some indication that studies exploring the functional form of their continuous predictors (either assessing the linearity assumption or using methods to handle nonlinear predictors) were more methodologically robust or more likely to follow good and established practices in model development and evaluation. For example, these studies were typically larger, more likely to use resampling methods to internally validate their models, and cited reporting their studies following the TRIPOD reporting guideline. These studies were also more likely to report the recommended measures to assess calibration (i.e., a calibration plot, calibration slope, and calibration-in-the-large). This suggests that researchers of studies exploring functional form had a better understanding and thought-out design and analysis to develop their prediction model. A higher number of events per predictor were included in the studies that explored the functional form of their continuous predictors. However, we note that for many studies, there were at most one or two events per predictor. When the sample size or number of events is this small and used to develop a model, checking the linearity assumption is challenging, and adequately handling continuous, nonlinear predictors is even more challenging due to either convergence issues or the risk of overfitting the model which can also weaken the prediction performance of the model. In this case, study teams may be forced to compromise modeling the shape of the outcome-predictor relationship. Of course, this need not be the case through well-thought-out analyses informed by comprehensive sample size calculations.

Also, when higher order terms (e.g., splines or fractional polynomials) are included in the model, it introduces the possibility of interaction terms between predictors. The inclusion of interaction terms adds complexity to the model and increases the number of parameters needed to be estimated. It is important that interaction terms are also considered, starting at the design stage, and are checked and reported.

### 4.2 Context

Handling continuous predictors is an important issue for prediction model research and is also an important item in the formal risk of bias assessment of these studies (item 4.2: “Were continuous and categorical predictors handled appropriately?”) [61,62]. Poor or lack of handling of continuous predictors may result in biased coefficients and misspecified models that ultimately lead to inaccurate predictions and thus increase the risk of bias of a prediction model.

Many reviews have highlighted poor reporting and methodological concerns about how continuous predictors are handled in the methods (i.e., use of categorization or dichotomization) and how they are presented in the results (often not reported), in line with our findings [20,21,63,64]. For example, a systematic review of prediction models for type two diabetes showed 63% of studies categorized all or some of continuous risk predictors, only 13% of studies considered nonlinear terms, and only one included nonlinear terms in the final model [47]. However, few studies have provided details about prevalence of studies assessing the nonlinearity assumption, how this is done, and how nonlinearity terms are reported in the results. These reviews also observed inadequate sample sizes when developing prediction models based on the number of candidate predictors and the number of available events [20,63,64]. While new sample size guidance is now available [13], it is highly unlikely that these studies would have met the new criteria.

Categorization, and in particular dichotomization (coined “dichotomania” by Stephen Senn [65]), has been a long-standing problem in regression modeling and has been warned against by statisticians [7,66]. Accurately predicting outcomes is challenging, and categorization, particularly with fewer categories, makes this prediction more difficult by discarding information, and while more categories might lose less information, the sample size requirements increase (more parameters need to be estimated). It also forces individuals with values above and below a cut point who are similar to have a different risk, and those in the same category (but at the extremes) will have the same risk but could be quite different. Assumptions of linearity should be checked and reported (e.g., residuals, model fit, plots) by the shape of the relationship between a continuous predictor and the outcome.

### 4.3 Strengths and limitations

Our review highlights current practice of handling continuous predictors in studies developing clinical prediction models, including assessment of the linearity assumption for regression modeling, methods to handle nonlinear predictors, and reporting nonlinear terms in the final model. We limited our search to studies published in a single electronic medical literature database and those published between July 01, 2020, and July 30, 2020. We used this sample of studies to both estimate the proportion of studies that assess the linearity assumption and describe how continuous predictors were commonly handled. It is unlikely that additional studies would change the conclusion of this review. A further limitation is that the search was carried out 3 years ago, and the study was stalled during the COVID-19 pandemic. However, given the long-standing concerns of handling continuous predictors (also observed in the large number of COVID-19 prediction models [67], contributing to the high risk of bias concerns) and no major initiatives in the intervening period to tackle this, including a more recent sample of papers, it is unlikely that this will have changed our results and conclusions.

We focused our review to studies predicting a binary outcome using logistic regression to reflect more common clinical prediction model scenarios and excluded studies predicting other outcome types (e.g., time to event), other modeling approaches (e.g., Cox regression), and nonlinear models of the predictor parameters themselves, where the functional form of continuous predictors would also need to be explored. However, given that studies have shown that the handling of continuous predictors is poor [68], irrespective of outcome type and modeling approaches, the findings of this review remain relevant and applicable.

Methods to handle nonlinear continuous predictors such as fractional polynomials and restricted cubic splines are available and widely implemented in statistical software (e.g., in R, Stata) but remain underused and poorly reported. Reasons for this might include a lack of involving statistical expertise, a lack of data, or the complexity of the functional form that is difficult to capture and present. For example, one study in our review reported that the "complex nonlinear relationships between the covariates and the outcome that are difficult to explicitly capture even with the use of techniques such as including polynomial terms or cubic spline" [41], and another study in our review reported that “the calculation of continuous variables is not simple and cannot be conducted mentally; therefore, we further simplified the model” [38].

The functional form can be complicated when reporting the regression coefficients, particularly when using restricted cubic splines (Supplementary Box 2), especially if the study has many continuous predictors. However, reporting of “complex” prediction models that include nonlinear terms is less of an issue with options to now make statistical code available (e.g., GitHub and the Open Science Framework). We have provided a list of R packages (Supplementary Table 5) that can be used for handling continuous predictors.

### 4.4 Future research/research recommendations

Handling continuous predictors should be considered prior to any analysis during study design and protocol development. Further guidance is needed to help researchers planning their research so that important study design features, such as sample size and handling of continuous predictors, are fully considered and accounted for. The STRengthening Analytical Thinking for Observational Studies initiative, and in particular topic group 2, provides evidence-supported guidance to researchers with a basic level of statistical knowledge on selection of functional forms in multivariable analyses [17,69]. The TRIPOD statement also explains why and how continuous predictors should be checked for linearity and how the handling of each predictor in the analysis should be clearly reported. Though TRIPOD is available as a reporting guideline for prediction model studies, additional guidance on appropriate methods for handling nonlinear predictors and reporting nonlinear terms in the final is needed. We have provided some recommendations (Box 2) on how to handle continuous predictors and what to report when developing a clinical prediction model. For example, if using restricted cubic splines, the number of knots and their location need to be reported for transparent research.

Box 1 **Description of common approaches to explore the functional form between a continuous predictor and the outcome11For binary outcomes, this is on the log-odds scale. to be predicted**

**Linear** is when a one-unit increase in the continuous predictor leads to a constant increase in the outcome across the whole range of the predictor values.

**Transformations** are simple mathematical operations, such as log, square root, and inverse, that are applied to a continuous predictor (all values) so that the (linear) association of the transformed predictor and the outcome is then modeled.

**Fractional polynomials** are a set of flexible power transformations to model the relationship between a continuous predictor and the outcome [31]. The class of fractional polynomials is defined by a set of eight power transformations (which includes fractional and negative transformations) including x^−2^, x^−1^, x^−0.5^, log(x), x^0.5^, x, x^2^, and x^3^. The transformations are combined into simple functions that best capture the relationship between the predictor and the outcome. A function selection procedure is used to identify the best-fitting function, comparing against a “default” linear function [32].

**Cubic splines** are piecewise cubic polynomials, where the continuous predictor values are subdivided by knots (cut points), and separate cubic polynomials are fit to the points that lie between the knots [15]. The polynomials are forced to meet at the knots to ensure a smooth relationship between the predictor and the outcome. Cubic splines can often fit poorly in the tails, so ***restricted cubic splines*** may be used, where before the first and after the last knot, the splines are restricted to be linear. The number and location of knots needs to be specified, with three to five knots often suitable, typically defined by quantiles of the continuous predictor (to ensure enough observations between knot locations for each cubic polynomial).

Box 2 **Recommendations on handling continuous predictors**

1. Protocol development
  a. Anticipate any potential nonlinear continuous predictor-outcome relationships during the study design.
  b. Account for any potential nonlinear parameters when calculating the sample size, that is, including additional predictor parameters in sample size calculation [12,14].
  c. Describe methods to assess the functional form and handling of potential nonlinear continuous predictors during the model building.
2. Avoid categorization or dichotomization of continuous predictors
  a. Continuous predictors are often converted into categorical or dichotomous variables [[20], [21], [22]], often to avoid making assumptions about the predictor-outcome relationship. The perceived reasoning behind categorization is clinical relevance, ease, and interpretability. However, categorizing continuous predictors imposes an implausible step function at the cut point, discards information, and comes at a loss in predictive accuracy [8,65].
3. Assess the functional form of each continuous predictor-outcome relationship
  a. Plot and visually assess the continuous predictor values against the log-odds (for binary)/log-hazard (for time to event) of the outcome and plots of deviance residuals after fitting linear and nonlinear terms.
  b. Appropriately model the functional form and report the methods (and details) used, for example, linear, transformations, restricted cubic splines (including the number and location of knots), and fractional polynomials.
4. Completely and transparently report all methods used to check and model the functional form of nonlinear predictors
  a. Use the TRIPOD statement to guide to reporting [18].
  b. Fully report the final developed model with all terms and the respective coefficient values, including the intercept. See Supplementary Box 2 for converting the terms using restricted cubic splines.

## 5 Conclusion

The handling of continuous predictors when developing a clinical prediction model is generally poor. Many studies are seemingly unaware or overlook the importance of correctly specifying the functional form of the relationship between the predictors and the outcome. Assuming linearity without checking and categorizing (and in particular dichotomizing) predictors can lead to models with poor predictive accuracy and, more importantly, poor predictions that could influence clinical decision-making and ultimately patient outcomes. While the importance of handling continuous predictors is widely understood among some researchers, there is clearly a need to provide guidance to the wider group of researchers who often carry out this research.

## CRediT authorship contribution statement

**Jie Ma:** Conceptualization, Methodology, Investigation, Data curation, Formal analysis, Writing – original draft, Writing – review & editing. **Paula Dhiman:** Conceptualization, Methodology, Investigation, Writing – review & editing, Supervision. **Cathy Qi:** Investigation, Writing – review & editing. **Garrett Bullock:** Investigation, Writing – review & editing. **Maarten van Smeden:** Conceptualization, Methodology, Writing – review & editing. **Richard D. Riley:** Conceptualization, Methodology, Writing – review & editing. **Gary S. Collins:** Conceptualization, Methodology, Writing – review & editing, Supervision.

## Declaration of Competing Interest

The authors of this manuscript have no conflicts of interest to declare.
