# Supplementary material for: Poor handling of continuous predictors in clinical prediction models using logistic regression: a systematic review
Source: J Clin Epidemiol. 2023 Sep;161:140–51. doi: 10.1016/j.jclinepi.2023.07.017 (PMC11913776; doi:10.1016/j.jclinepi.2023.07.017)
Supplement: Supplementary [file mmc1.docx]

**Supplementary material**

**Supplementary Box 1. Search string**

| The final PubMed search strategy is:  ('prediction'[tiab] OR 'predictive'[tiab] OR 'risk' [tiab] OR 'prognostic'[tiab] OR 'diagnostic'[tiab] 'discrimination'[tiab] OR 'calibration'[tiab] OR 'area under the curve'[tiab] OR 'auc'[tiab] OR 'c-statistic'[tiab] OR 'ROC'[tiab] OR 'area under ROC curve '[tiab] OR 'auROC[tiab] OR 'area under the receiver operating characteristic curve'[tiab] OR 'area under receiver operating characteristic curve'[tiab]) AND ('model'[tiab] OR 'equation'[tiab] OR calculator'[tiab] OR 'assessment'[tiab] OR 'decision making'[tiab] OR 'rule'[tiab] OR 'score'[tiab] OR 'risk model'[tiab] OR 'statistical model'[tiab] OR 'multivariable'[tiab] OR 'statistical regression'[tiab] OR 'risk score'[tiab] OR 'logistic'[tiab] OR 'cox'[tiab] OR 'survival'[tiab] OR 'regression'[tiab]) NOT ('Radiomics'[tiab] OR 'miRNAs'[tiab] OR 'mRNA'[tiab] OR 'lncRNAs'[tiab] OR 'gene'[tiab] OR 'radio'[tiab] OR 'cell'[tiab] OR 'systematic review'[tiab] OR review[Publication Type] OR Bibliography[Publication Type] OR Editorial[Publication Type] OR Letter[Publication Type] OR Meta-analysis[Publication Type] OR News[Publication Type])  Final search executed on 03/08/2020. |
| --- |

**Supplementary Box 2. Example of how to write down a restricted cubic spline**

Let k knots be placed at t_k_, then

$$y_{i}=\beta_{0}+\beta_{1}x_{i}+\beta_{2}x_{i}^{2}+\beta_{3}x_{i}^{3}+\varepsilon_{i} if x_{i}\in t_{k}$$

for a continuous predictor x, a set of (k-2) new variables are created:

$$x_{i}={(x-t_{i})}_{+}^{3}-\left( x-t_{k-1} \right)_{+}^{3}\frac{t_{k}-t_{i}}{t_{k}-t_{k-1}}+\left( x-t_{k} \right)_{+}^{3}\frac{t_{k-1}-t_{i}}{t_{k}-t_{k-1}},i=1,\ldots,k-2$$

Then, the original continuous predictor has been augmented by this set of new variables, each of which is linear in the regression coefficients. The model can therefore be fit using the usual regression procedures, and inferences can be drawn as usual.

Cubic spines are easy to use with any statistical program (e.g., rcs function for restricted cubic spline in R) but the interpretation for individual terms was not suggested (*Regression Modelling Strategies, Harrell*).

**Supplementary table 1. Full reference list of included studies**

| **Author** | **title** | **Clinical specialty** | **Functional form explored** |
| --- | --- | --- | --- |
| Jalali[1] | A risk calculator to inform the need for a prostate biopsy: a rapid access clinic cohort | Oncology | Yes |
| Milton[2] | ICU discharge screening for prediction of new-onset physical disability-A multinational cohort study | Intensive care medicine | Yes |
| Song[3] | Prediction model for clinical pregnancy for ICSI after surgical sperm retrieval in different types of azoospermia | Obstetrics and gynecology | Yes |
| Xiao[4] | Models for acute on chronic liver failure development and mortality in a veterans affairs cohort | Hepatology | Yes |
| Kong[5] | A Simple Four-factor Preoperative Recipient Scoring Model for Prediction of 90-day Mortality after Adult Liver Transplantation: A Retrospective Cohort Study | Hepatology | Yes |
| Alqahtani[6] | Can we better predict readmission for dehydration following creation of a diverting loop ileostomy: development and validation of a prediction model and web-based risk calculator | Colorectal surgery | Yes |
| Bronsert[7] | Identification of postoperative complications using electronic health record data and machine learning | General surgery | Yes |
| Mourelo-Fariña[8] | A Model for Prediction of In-Hospital Mortality in Patients with Subarachnoid Hemorrhage | Neurology | Yes |
| Johnsen[9] | Development and validation of a prediction model for incident hand osteoarthritis in the HUNT study | Rheumatology | Yes |
| Pareek[10] | A practical risk score for early prediction of neurological outcome after out-of-hospital cardiac arrest: MIRACLE2 | Emergency medicine | Yes |
| Zelis[11] | A new simplified model for predicting 30-day mortality in older medical emergency department patients: The rise up score | Emergency medicine | Yes |
| Roshanov[12] | Preoperative prediction of Bleeding Independently associated with Mortality after noncardiac Surgery (BIMS): an international prospective cohort study | General surgery | Yes |
| Sutradhar[13] | Comparing an Artificial Neural Network to Logistic Regression for Predicting ED Visit Risk Among Patients With Cancer: A Population-Based Cohort Study | Oncology | Yes |
| Kiddle[14] | Prediction of five-year mortality after COPD diagnosis using primary care records | Respiratory medicine | Yes |
| Willems[15] | Baseline Patient Characteristics Commonly Captured Before Surgery Do Not Accurately Predict Long-Term Outcomes of Lumbar Microdiscectomy Followed by Physiotherapy | Spinal surgery | Yes |
| Nguyen[16] | Interval breast cancer risk associations with breast density, family history and breast tissue aging | Oncology | Yes |
| Bai[17] | A Predictive Model for the Identification of Cardiac Effusions Misclassified by Light's Criteria | Pulmonology | Yes |
| Raita[18] | Machine learning-based prediction of acute severity in infants hospitalized for bronchiolitis: a multicenter prospective study | Pediatrics | Yes |
|  |  |  |  |
|  |  |  |  |
| Abujaber[19] | Using trauma registry data to predict prolonged mechanical ventilation in patients with traumatic brain injury: Machine learning approach | Neurology | No |
| Knoepfel[20] | The AdHOC (age, head injury, oxygenation, circulation) score: a simple assessment tool for early assessment of severely injured patients with major fractures | Trauma and Orthopaedics | No |
| Merhe[21] | Development of a novel nomogram incorporating platelet-to-lymphocyte ratio for the prediction of lymph node involvement in prostate carcinoma | Urology | No |
| Roposch[22] | Predicting developmental dysplasia of the hip in at-risk newborns | Pediatrics | No |
| Togawa[23] | Development of risk factor-based scoring system for detection of hypervirulent Klebsiella pneumoniae bloodstream infections | Pulmonology | No |
| Zeadna[24] | Prediction of sperm extraction in non-obstructive azoospermia patients: a machine-learning perspective | Urology | No |
| Anderson[25] | Can Predictive Modeling Tools Identify Patients at High Risk of Prolonged Opioid Use After ACL Reconstruction? | Orthopaedics | No |
| Kao[26] | The CELIOtomy Risk Score: An effort to minimize futile surgery with analysis of early postoperative mortality after emergency laparotomy | General surgery | No |
| Chen[27] | Nomogram to predict postpartum hemorrhage in cesarean delivery for women with scarred uterus: A retrospective cohort study in China | Obstetrics and gynecology | No |
| Duceau[28] | Prehospital triage of acute aortic syndrome using a machine learning algorithm | Emergency medicine | No |
| Yang[29] | Large population-based study using the SEER database: is endoscopic resection appropriate for early gastric cancer patients in the United States? | Oncology | No |
| Zhao[30] | Predictive Model for Pulmonary Embolism in Patients with Deep Vein Thrombosis | Vascular surgery | No |
| Bender[31] | Assessment of Simple Bedside Wound Characteristics for a Prediction Model for Diabetic Foot Ulcer Outcomes | Endocrinology | No |
| Chattot[32] | Preoperative predictors and a prediction score for perception of improvement after mesh prolapse surgery | Obstetrics and gynecology | No |
| Han[33] | Early morning off in patients with Parkinsons disease: a Chinese nationwide study and a 7-question screening scale | Neurology | No |
| Cotton[34] | A Model Using Clinical and Endoscopic Characteristics Identifies Patients at Risk for Eosinophilic Esophagitis According to Updated Diagnostic Guidelines | Gastroenterology | No |
| Monamele[35] | Clinical signs predictive of influenza virus infection in Cameroon | Respiratory | No |
| Chen[36] | Check point to get adequate weight loss within 6-months after laparoscopic sleeve gastrectomy for morbid obesity in Asian population | Gastroenterology | No |
| Tsilimigras[37] | Very Early Recurrence After Liver Resection for Intrahepatic Cholangiocarcinoma: Considering Alternative Treatment Approaches | Oncology | No |
| Jiang[38] | Predicting and comparing postoperative infections in different stratification following PCNL based on nomograms | Nephrology | No |
| Vesale[39] | Predictive approach in managing voiding dysfunction after surgery for deep endometriosis: a personalized nomogram | Gastroenterology | No |
| Capretti[40] | Enhanced Recovery After Pancreatic Surgery Does One Size Really Fit All? A Clinical Score to Predict the Failure of an Enhanced Recovery Protocol After Pancreaticoduodenectomy | Hepatobiliary surgery | No |
| Gök[41] | A New Risk Score to Predict In-Hospital Mortality in Elderly Patients With Acute Heart Failure: On Behalf of the Journey HF-TR Study Investigators | Cardiology | No |
| Wong[42] | Refeeding Hypophosphatemia in Patients Receiving Parenteral Nutrition: Prevalence, Risk Factors and Predicting Its Occurrence | Gastroenterology | No |
| Lian[43] | Early prediction of cerebral-cardiac syndrome after ischemic stroke: the PANSCAN scale | Neurology | No |
| Muttai[44] | Development and Validation of a Sociodemographic and Behavioral Characteristics-Based Risk-Score Algorithm for Targeting HIV Testing Among Adults in Kenya | Sexual Health | No |
| Foroushani[45] | Quantitative Serial CT Imaging-Derived Features Improve Prediction of Malignant Cerebral Edema after Ischemic Stroke | Neurology | No |
| Kwak[46] | Can Computed Tomographic Angiography Be Used to Predict Who Will Not Benefit from Endovascular Treatment in Patients with Acute Ischemic Stroke? The CTA-ABC Score | Neurosurgery | No |
| Gagnon[47] | Parent-Child Agreement on Postconcussion Symptoms in the Acute Postinjury Period | Pediatrics | No |
| Niyongombwa[48] | Kigali Surgical Sepsis (KiSS) Score: A New Tool to Predict Outcomes in Surgical Patients with Sepsis in Low- and Middle-Income Settings | General surgery | No |
| Tseng[49] | Predictors of Acute Mortality After Open Pelvic Fracture: Experience From 37 Patients From A Level I Trauma Center | Trauma and Orthopaedics | No |
| Ellis-Kahana[50] | Developing a model for predicting venous thromboembolism in obese pregnant women in a national study | Obstetrics and gynecology | No |
| Feghali[51] | Novel Risk Calculator for Suboccipital Decompression for Adult Chiari Malformation | Neurosurgery | No |
| Ma[52] | Development and validation of a risk stratification model for screening suspected cases of COVID-19 in China | Respiratory | No |
| Vivier-Chicoteau[53] | Development and internal validation of a diagnostic score for gastric linitis plastica | Oncology | No |
| Xiao[54] | Discriminating Malignancy in Thyroid Nodules: The Nomogram Versus the Kwak and ACR TI-RADS | Oncology | No |
| Sim[55] | The major effects of health-related quality of life on 5-year survival prediction among lung cancer survivors: applications of machine learning | Oncology | No |
| Mejia-Otero[56] | Risk factors for hospitalization in youth with type 1 diabetes: Development and validation of a multivariable prediction model | Endocrinology | No |
| Delparte[57] | Development of the spinal cord injury pressure sore onset risk screening (SCI-PreSORS) instrument: a pressure injury risk decision tree for spinal cord injury rehabilitation | Physical medicine and rehabilitation | No |
| Shoenbill[58] | Identifying patterns and predictors of lifestyle modification in electronic health record documentation using statistical and machine learning methods | General medicine | No |
| Uchida[59] | Simplified Prehospital Prediction Rule to Estimate the Likelihood of 4 Types of Stroke: The 7-Item Japan Urgent Stroke Triage (JUST-7) Score | Neurology | No |
| Rothenberg[60] | Assessment of the Risk Analysis Index for Prediction of Mortality, Major Complications, and Length of Stay in Patients who Underwent Vascular Surgery | Vascular surgery | No |
| Benoit[61] | Nomogram Predicting the Likelihood of Parametrial Involvement in Early-Stage Cervical Cancer: Avoiding Unjustified Radical Hysterectomies | Oncology | No |
| Qin[62] | A predictive model and scoring system combining clinical and CT characteristics for the diagnosis of COVID-19 | Respiratory | No |
| Wu[63] | Predicting treatment failure risk in a Chinese Drug-Resistant Tuberculosis with surgical therapy: Development and assessment of a new predictive nomogram | Respiratory | No |
| Zhan[64] | Nomogram Model for Predicting Risk of Postoperative Delirium After Deep Brain Stimulation Surgery in Patients Older Than 50 Years with Parkinson Disease | Neurology | No |
| Rocio[65] | Interleukin-6-based mortality risk model for hospitalised COVID-19 patients | Respiratory | No |
| Tennenhouse[66] | Machine-learning models for depression and anxiety in individuals with immune-mediated inflammatory disease | Immunology | No |
| Vitzthum[67] | Predicting Persistent Opioid Use Abuse and Toxicity Among Cancer Survivors | Oncology | No |
| Xiao[68] | Development and validation of the HNC-LL score for predicting the severity of coronavirus disease 2019 | Respiratory | No |
| Ho-Pham[69] | Development of a model for identification of individuals with high risk of osteoporosis | Rheumatology | No |
| Yin[70] | An internally validated diagnostic tool for acute invasive fungal sinusitis International forum of allergy & rhinology. | Otorhinolaryngology | No |
| Baimas-George[71] | A pre-operative platelet transfusion algorithm for patients with cirrhosis and hepatocellular carcinoma undergoing laparoscopic microwave ablation | Oncology | No |
| Iriondo[72] | Prediction of mortality in very low birth weight neonates in Spain | Pediatrics | No |
| Jacob[73] | Predicting lung nodules malignancy | Oncology | No |
| Ladios-Martin[74] | Predictive Modeling of Pressure Injury Risk in Patients Admitted to an Intensive Care Unit | Intensive care medicine | No |
| Raseta[75] | A novel toolkit for the prediction of clinical outcomes following mechanical thrombectomy | Neurology | No |
| Tago[76] | New predictive models for falls among inpatients using public ADL scale in Japan: A retrospective observational study of 7858 patients in acute care setting | General medicine | No |
| Xue[77] | A nomogram model for screening the risk of diabetes in a large-scale Chinese population: an observational study from 345718 participants | Endocrinology | No |
| Yamamoto[78] | Clinical risk model for predicting 1-year mortality after transcatheter aortic valve replacement | Cardiology | No |
| Zhang[79] | Gastrointestinal bleeding in patients admitted to cardiology: risk factors and a new risk score | Cardiology | No |
| Naim[80] | Development and Validation of a Seizure Prediction Model in Neonates Following Cardiac Surgery | Pediatric surgery | No |
| Balachandren[81] | Ovarian reserve as a predictor of cumulative live birth | Obstetrics and gynecology | No |
| Hou[82] | Development and Validation of a Nomogram for Individually Predicting Pathologic Complete Remission After Preoperative Chemotherapy in Chinese Breast Cancer: A Population-Based Study | Oncology | No |
| Zhang[83] | Use of Nutrition Risk in Critically ill (NUTRIC) scoring system for nutritional risk assessment and prognosis prediction in critically ill neurological patients: a prospective observational study | Neurology | No |
| Zhang[84] | Risk factors analysis and a nomogram model establishment for late postoperative seizures in patients with meningioma | Oncology | No |
| Tseng[85] | Prediction of the development of acute kidney injury following cardiac surgery by machine learning Critical care (London England) | Cardiology | No |
| Wu[86] | Efficacy of interleukin-6 in combination with D-dimer in predicting early poor postoperative prognosis after acute stanford type a aortic dissection | vascular surgery | No |
| Chu[87] | Predicting the Risk of Adverse Events in Pregnant Women With Congenital Heart Disease | Cardiology | No |
| Yamamoto[88] | Modified abbreviated burn severity index as a predictor of in-hospital mortality in patients with inhalation injury: development and validation using independent cohorts | Respiratory | No |
| Benirschke[89] | Detection of Falsely Elevated Point-of-Care Potassium Results Due to Hemolysis Using Predictive Analytics | Secondary care | No |
| Luzzago[90] | A novel nomogram to identify candidates for active surveillance amongst patients with International Society of Urological Pathology (ISUP) Grade Group (GG) 1 or ISUP GG2 prostate cancer according to multiparametric magnetic resonance imaging findings | Oncology | No |
| Wang[91] | Development and validation of a novel scoring system developed from a nomogram to identify malignant pleural effusion | Respiratory | No |
| Klim[92] | Combined serum biomarker analysis shows no benefit in the diagnosis of periprosthetic joint infection | Orthopaedics | No |
| Kojima[93] | Keratoconus Screening Using Values Derived From Auto-Keratometer Measurements: A Multicenter Study | Ophthalmology | No |
| Vieceli[94] | A predictive score for COVID-19 diagnosis using clinical laboratory and chest image data | Respiratory | No |
| Kalhan[95] | Caries Risk Prediction Models in a Medical Health Care Setting | Pediatrics | No |
| Puar[96] | Aldosterone-potassium ratio predicts primary aldosteronism subtype | Endocrine surgery | No |
| Kuo[97] | Nomogram for pneumonia prediction among children and young people with cerebral palsy: A population-based cohort study | Pediatrics | No |
| Sun[98] | Development and validation of two aspiration prediction models in patients receiving nasogastric feeding | Gastroenterology | No |
| Wang[99] | A nomogram to predict skip metastasis in papillary thyroid cancer | Oncology | No |
| Chung[100] | Novel mechanical ventilator weaning predictive model | Intensive care medicine | No |
| Zheng[101] | Establishment and internal validation of preoperative nomograms for predicting the possibility of testicular salvage in patients with testicular torsion | Urology | No |
| Du[102] | Predicting in-hospital mortality of patients with febrile neutropenia using machine learning models | Oncology | No |
| Li[103] | A Novel Risk Stratification Score for Sudden Cardiac Death Prediction in Middle-Aged Nonischemic Dilated Cardiomyopathy Patients: The ESTIMATED Score | Cardiology | No |
| Pan[104] | Risk Prediction for Non-alcoholic Fatty Liver Disease Based on Biochemical and Dietary Variables in a Chinese Han Population Frontiers in public health | Hepatology | No |
| Tang[105] | Predicting poor response to neoadjuvant chemoradiotherapy for locally advanced rectal cancer: Model constructed using pre-treatment MRI features of structured report template | Oncology | No |
| Chen[106] | A Novel Prediction Model for Significant Liver Fibrosis in Patients with Chronic Hepatitis B | Hepatology | No |
| Fan[107] | Distinction and Potential Prediction of Lung Metastasis in Patients with Malignant Primary Osseous Spinal Neoplasms | Oncology | No |
| Gu[108] | Is Cervical Traction Effective in Chronic Nonspecific Neck Pain Patients With Unsatisfactory NSAID Control? A Nomogram to Predict Effectiveness | Spinal | No |
| Shimizu[109] | New Model for Predicting Malignancy in Patients With Intraductal Papillary Mucinous Neoplasm | Oncology | No |
| Sun[110] | Epidemiological and Clinical Predictors of COVID-19 | Respiratory | No |
| Zhao[111] | Application of data mining for predicting hemodynamics instability during pheochromocytoma surgery | Endocrine surgery | No |
| Zhang[112] | Establishment of a Risk Prediction Model for Non-alcoholic Fatty Liver Disease in Type 2 Diabetes | Endocrinology | No |
| Cai[113] | A Novel Nomogram Predicting Distant Metastasis in T1 and T2 Gallbladder Cancer: A SEER-based Study | Oncology | No |
| Zhang[114] | A mid-pregnancy risk prediction model for gestational diabetes mellitus based on the maternal status in combination with ultrasound and serological findings | Obstetrics and gynecology | No |
| Du[115] | Accurate Prediction of Coronary Heart Disease for Patients With Hypertension From Electronic Health Records With Big Data and Machine-Learning Methods: Model Development and Performance Evaluation | Cardiology | No |
| Wang[116] | Nomogram Analysis and Internal Validation to Predict the Risk of Cystobiliary Communication in Patients Undergoing Hydatid Liver Cyst Surgery | Hepatobiliary surgery | No |
| Zhao[117] | Prediction model and risk scores of ICU admission and mortality in COVID-19 | Respiratory | No |
| Lv[118] | Construction of a Risk Prediction Model for Fever After Painless Bronchoscopy | Respiratory | No |

**Supplementary table 2. Model and non-linear term presentation**

| 1 | Models for acute on chronic liver failure development and mortality in a veterans affairs cohort | INR_spline1: 3.1e+19,INR_spline2: 0.00;Total bilirubin_spline1: 313.60, Total bilirubin_spline2: 0.00; Creatinine_spline1:0.11, Creatinine_spline2: 9.33; Albumin_spline1: 0.53, Albumin_spline2: 1.41; Hemoglobin_spline1: 0.80, Hemoglobin_spline2: 1.10; coeffictients listed in supplemental table | Methods: For continuous variables, we evaluated the linearity assumption against the outcome of interest using locally weighted scatterplot smoothing (LOWESS) plots. If there was notable non-linearity, we used restricted cubic splines. The initial number and location of knots were selected at Harrell’s recommended percentiles. If ongoing deviation was evident between the LOWESS curve fitted to raw data and the spline model by visual inspection, we refined the model by modifying a knot value in the area of deviation or by adding a knot. We prioritized achieving agreement between these curves while minimizing the numberof knots used. Final knot selections are shown in Supplemental Table 1.  Results: NR  Discussion: NR |
| --- | --- | --- | --- |
| 2 | A Predictive Model for the Identification of Cardiac Effusions Misclassified by Light's Criteria | −4.31log10 (PF − LDH) + 1.01log10 (P/S LDH)−1.94log10 (PF − ADA)+2.53log10 (NT − pro − BNP) | **Methods**: The PF-LDH, P/S LDH, PF-ADA, and NT-pro-BNP were log transformed, as these variables showed skewed distributions.  **Results**: NR  **Discussion**: NR |
| 3 | A risk calculator to inform the need for a prostate biopsy: a rapid access clinic cohort | LogPSA | **Methods**: The Irish Prostate Cancer Risk Calculator (IPRC) for the diagnosis of PCa (and high-grade PCa) is built for the Irish population on the total cohort including linear and non-linear effects of components such as age, digital rectal examination, family history of PCa, prior negative biopsy and PSA level.  **Results**: NR  **Discussion**: NR |
| 4 | Preoperative prediction of Bleeding Independently associated with Mortality after noncardiac Surgery (BIMS): an international prospective cohort study | full equation in supp; -0.0102131 * eGFR in ml min-1 1.73 m-2 + 2.564964×10^−6 * MAX(eGFR in ml min-1 1.73 m-2 – 47, 0)^3 − 6.962045×10^−6 * MAX(eGFR in ml min-1 1.73 m-2 – 83, 0)^3 + 4.397081×10^−6 * MAX(eGFR in ml min-1 1.73 m-2 – 104, 0)^3 − 0.0630985 * preoperative haemoglobin in g L-1 + 7.77466×10^−6 * MAX(preoperative haemoglobin in g L-1 – 105, 0)^3 + −1.865918×10^−5 * MAX(preoperative haemoglobin in g L-1 – 133, 0)^3 + 1.088452×10^−5 * (preoperative haemoglobin in g L-1 – 153, 0)^3 | **Methods:** We modelled continuous variables (age, preoperative haemoglobin, and eGFR) using restricted cubic spline functions to allow for non-linearity in their relationship with BIMS.  **Results**: The relationship between preoperative haemoglobin and BIMS (Supplementary Fig. S1a) was nonlinear (P-value for non-linearity <0.001), with the risk of BIMS increasing more rapidly; The relationship between kidney function (eGFR) and BIMS was also non-linear (P-value for nonlinearity <0.001), with the risk of BIMS only increasing with eGFR<80 ml min^-1 1.73m^-2 (Supplementary Fig. S1b). The risk of BIMS increased linearly with age (P-value for non-linearly=0.90.  **Discussion**: NR |
| 5 | A new simplified model for predicting 30-day mortality in older medical emergency department patients: The rise up score | Only dichotomised: Bilirubin >20 μmol/L | **Method**: Linearity was visually checked for all possible continuous predictors and if necessary, continuous predictors were log transformed or dichotomised, based on their relationship with the outcome.  **Results**: NR  **Discussion**: NR |
| 6 | Prediction model for clinical pregnancy for ICSI after surgical sperm retrieval in different types of azoospermia | Not included | **Methods**: quadratic terms were not statistically significant.  **Results**: NR  **Discussion**: NR  **supplementary**: Two (first-order) or three (quadratic) covariables were multiplied to form a new variable, and the new variable was put into the prediction model, and multivariate logistic regression was used to analyse whether the new variable is associated with clinical pregnancy. |
| 7 | ICU discharge screening for prediction of new-onset physical disability—A multinational cohort study | Not included | **Method**: Relationship for non-linear trends was tested by introducing the continuous predictors in logistic regression models by means of three-knot natural cubic splines.  **Results**: NR  **Discussion**: NR |
| 8 | Machine learning‑based prediction of acute severity in infants hospitalized for bronchiolitis: a multicenter prospective study | No final model details | **Methods**: First, we investigated non-linear relationships between the continuous predictors and outcomes  and created quadric terms of age, respiratory rate, and temperature. These quadratic terms were used only for  regression-based machine learning models (i.e., logistic regression models with Lasso regularization and those  with elastic net regularization).  **Results**: NR  **Discussion**: NR |
| 9 | Baseline Patient Characteristics Commonly Captured Before Surgery Do Not Accurately Predict Long-Term Outcomes of Lumbar Microdiscectomy Followed by Physiotherapy | Not included | **Methods**: All assumptions (linearity between independent continuous variables, log odds, and multicollinearity) were checked before model building.  **Results**: NR  **Discussion**: NR |
| 10 | A practical risk score for early prediction of neurological outcome after out-of-hospital cardiac arrest: MIRACLE2 | Age category: <60, 60-80, >80; pH<7.20 | **Method**: However, variables with a strong or non-linear association could be assigned extra points, where this does not affect practicality of use.  **Results:** Continuous variables (age and pH) were also categorized. A value of 7.20 was used for pH and, since age showed a quadratic relationship with the outcome, three age categories were used with cut-off points at 60 years (one point) and 80 years for age.  **Discussion**: NR |
| 11 | Prediction of five-year mortality after COPD diagnosis using primary care records | Age in years from 67.7, Age in years from 67.7, squared; Body Mass Index in kg/m2 from 26, Body Mass Index in kg/m2 from 26, squared; FEV1% predicted from 64.6%, FEV1% predicted from 64.6%, squared | NR |
| 12 | Identification of postoperative complications using electronic health record data and machine learning | Included as linear | **Methods:** NR  **Results**: All variables were dichotomous, except for CPT-specific complication event rate, which was continuous. The relationship between CPT specific complication event rate and the probability of any complication was approximately linear when visually inspected using a cubic smoothing spline.  **Discussion**: NR |
| 13 | Can we better predict readmission for dehydration following creation of a diverting loop ileostomy: development and validation of a prediction model and web-based risk calculator | 0.002Opetime-0.00000013Opetime^2+0.002LOS-0.003LOS^2 | **Methods**: several functional forms (categorical, continuous and polynomial) were assessed for each candidate variable in a logistic regression model.  **Results**: NR  **Discussion**: NR |
| 14 | Interval breast cancer risk associations with breast density, family history and breast tissue aging | Adjusted breast density; Adjusted breast tissue aging | **Method**: For both breast density and breast tissue aging, a cube-root function gave the optimal Box–Cox power transformation to normality. Transformed breast density was adjusted for age and BMI, and transformed breast tissue aging was adjusted for age, using linear regression. **Results**: NR  **Discussion**: NR |
| 15 | A Model for Prediction of In-Hospital Mortality in Patients with Subarachnoid Hemorrhage | Not included | **Method**: Finally, the functional form of continuous predictors (linear vs. non-linear relationships) was assessed using restricted cubic splines, taking the 50th percentile as a reference point.  **Results**: NR  **Discussion**: NR |
| 16 | Comparing an Artificial Neural Network to Logistic Regression for Predicting ED Visit Risk Among Patients With Cancer: A Population-Based Cohort Study | Not included | **Method**: polynomial relationships for continuous covariates were also explored.  **Results:** NR  **Discussion**: "complex nonlinear relationships between the covariates and the outcome that are difficult to explicitly capture even with the use of techniques such as including polynomial terms or cubic spline"; |
| 17 | A simple four-factor preoperative recipient scoring model for prediction of T 90-day mortality after adult liver Transplantation:A retrospective cohort study | Unclear | **Methods:** NR  **Results**: Age did not exhibit a linear relationship. When the patient's age was>65 years, the mortality rate increased sharply.  **Discussion**: However, the calculation of continuous variables is not simple and cannot be conducted mentally; therefore, we further simplified the model |
| 18 | Development and validation of a prediction model for incident hand osteoarthritis in the HUNT study | 0.07 * age spline1+ (-0.05 * age spline2)+0.06 * age spline3 | **Methods**: Third, nonlinear effects of continuous variables were assessed using restricted cubic spline plots with the recommendations of Harrell regarding the number and location of knots. If deviation from linearity were observed, we created linear splines with knots based on the plot to estimate linear effects.  **Results**: For men, age was included as linear splines (knots at 50 and 60 years) and BMI were kept as continuous.  **Discussion:** We also used updated statistical methods, e.g., to assess nonlinear effects, using restricted cubic splines. When nonlinearity is present, splines represents a better and more flexible way to model a continuous predictor, i.e., due to reducing the loss of information from categorizing and potentially reducing the risk of over- or underestimation of the model. We acknowledge that splines may not be as easily interpretable as categorization. Therefore, age was also included as a categorical variable in the models, For men, age was included as linear splines (knots at 50 and 60 years) and BMI were kept as continuous.(supplementary) |

**Supplementary table 3. Validation method, model performance measures used in the study.**

|  | All studies (n=118) | Studies addressed non-linearity (n=18) | Studies did not address non-linearity (n=100) |
| --- | --- | --- | --- |
| Validation methods |  | | |
| Bootstrapping | 33 (30.0) | 8 (44.4) | 25 (25) |
| + cross validation | 2 (1.7) | 0 | 2 (3) |
| + temporal split sample | 3 (2.5) | 1 (5.5) | 2 (2) |
| Split sample | 44 (37.3) | 4 (22.2) | 40 (40) |
| + cross validation | 6 (5.1) | 3 (16.7) | 3 (3) |
| Cross-validation | 10 (8.5) | 1 (5.5) | 9 (9) |
| Development data | 11 (9.3) | 0 | 11 (11) |
| Unclear | 9 (7.6) | 1 (5.5) | 8 (8) |
| Performance measures |  | | |
| Discrimination | 116 (98.3) | 18 (100) | 98 (98) |
| Reported recommended calibration metrics* | 63 (52.5) | 13 (72.2) | 50 (49) |
| Other** | 84 (71.2) | 13 (72.2) | 71 (71) |

* Including a calibration plot or reporting the calibration slope or intercept

**Other performance includes sensitivity, specificity, negative predictive values, positive predictive values, accuracy, brier score, decision curve analysis, Youden index, precision, recall, F1 score

**Supplementary table 4.** Median apparent, split sample and bias-corrected c-statistics, with range and number of models informing the analysis, by method used to handle continuous predictors.

| **Method to handle continuous predictors** | **N (studies)** | **Median apparent c-statistic (range), n** | **Median split sample c-statistic (range), n** | **Median bias corrected c-statistic (range), n** |
| --- | --- | --- | --- | --- |
| Explored functional form | 18 | 0.84 (0.63 to 0.95)  12 models | 0.77 (0.73 to 0.9)  4 models | 0.83 (0.59 to 0.95) 11 models |
| Included nonlinear term in final model | 10 | 0.89 (0.76 to 0.95)  6 models | 0.8 (0.73 to 0.9)  3 models | 0.86 (0.67 to 0.95)  6 models |
| Did not explore functional form | 100 | 0.83 (0.68 to 0.99)  40 models | 0.81 (0.61 to 1,  39 models | 0.84 (0.63 to 0.99) 41 models |
| Categorised at least 1 continuous predictor* | 67 | 0.83 (0.65 to 0.99)  28 models | 0.82 (0.67 to 0.98) 27 models | 0.84 (0.68 to 0.96) 25 models |
| Categorised all continuous predictors** | 42 | 0.84 (0.65 to 0.97)  19 models | 0.81 (0.67 to 0.98)  15 models | 0.86 (0.68 to 0.92) 16 models |
| Assumed linearity of at least 1 continuous predictor*** | 61 | 0.82 (0.63 to 0.99)  23 models | 0.82 (0.61 to 1)  24 models | 0.83 (0.59 to 0.99) 27 models |
| Assumed linearity of all continuous predictors*** | 39 | 0.82 (0.63 to 0.99)  16 models | 0.79 (0.61 to 1)  14 models | 0.83 (0.59 to 0.99) 19 models |
| Assumed linearity and categorised continuous predictors | 22 | 0.82 (0.74 to 0.99)  7 models | 0.86 (0.69 to 0.98)  10 models | 0.84 (0.68 to 0.96)  8 models |

* includes 5 studies exploring functional form

** includes 2 studies exploring functional form

***includes 1 study exploring functional form

**Supplementary table 5.** R package used for method handling continuous predictors

| Package | Description | Author | webpage |
| --- | --- | --- | --- |
| rms | Regression modelling strategies. | Frank Harrell Jr | https://cran.r-project.org/package=rms |
| gss | A comprehensive package for structural multivariate function estimation using smoothing splines. | Chong Gu | https://cran.r-project.org/package=gss |
| pspline | Penalized smoothing splines. | Brian Ripley | https://cran.r-project.org/package=pspline |
| logspline | Log spline density estimation routines. | Charles Kooperberg | https://cran.r-project.org/package=logspline |
| splines2 | Regression spline functions and classes. | Wenjie Wang, Jun Yan | https://cran.r-project.org/package=splines2 |
| bigsplines | Fits smoothing spline regression models using scalable algorithms designed for large samples. | Nathaniel E. Helwig | https://cran.r-project.org/package=bigsplines |
| mfp | Fractional polynomials are used to represent curvature in regression models. | Georg Heinze, Gareth Ambler, Axel Benner | https://cran.r-project.org/package=mfp |

**
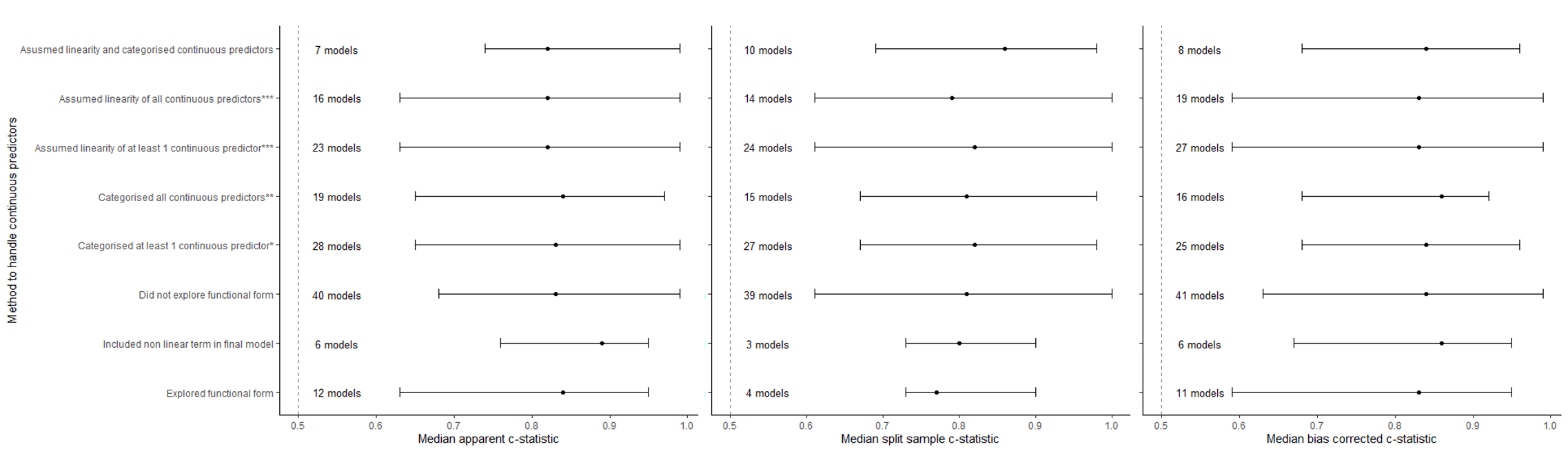
**

**Supplementary figure 1.** Forest plot of median apparent, split sample and bias-corrected c-statistics, with range and number of models informing the analysis, by method used to handle continuous predictors.

1. Jalali A, F.R., Maweni RM, Murphy K, et al., *A risk calculator to inform the need for a prostate biopsy: a rapid access clinic cohort.* BMC medical informatics and decision making, 2020. **20**(1): p. 148.

2. Milton A, S.A., Soliman I, et al., *ICU discharge screening for prediction of new-onset physical disability-A multinational cohort study.* Acta anaesthesiologica Scandinavica, 2020. **64**(6): p. 789-797.

3. Song J, G.L., et al., *Prediction model for clinical pregnancy for ICSI after surgical sperm retrieval in different types of azoospermia.* Human reproduction (Oxford, England), 2020.

4. Xiao KY, H.R., et al., *Models for acute on chronic liver failure development and mortality in a veterans affairs cohort.* Hepatology international, 2020. **14**(4): p. 587-596.

5. Kong L, L.T., et al., *A Simple Four-factor Preoperative Recipient Scoring Model for Prediction of 90-day Mortality after Adult Liver Transplantation:A Retrospective Cohort Study.* International journal of surgery (London, England), 2020.

6. Alqahtani M, G.R., et al., *Can we better predict readmission for dehydration following creation of a diverting loop ileostomy: development and validation of a prediction model and web-based risk calculator.* Surgical endoscopy, 2020. **34**(7): p. 3118-3125.

7. Bronsert M, S.A., et al., *Identification of postoperative complications using electronic health record data and machine learning.* American journal of surgery, 2020. **220**(1): p. 114-119.

8. Mourelo-Fariña M, P.S. and G. R, *A Model for Prediction of In-Hospital Mortality in Patients with Subarachnoid Hemorrhage.* Neurocritical care, 2020.

9. Johnsen MB, M.K., et al., *Development and validation of a prediction model for incident hand osteoarthritis in the HUNT study.* Osteoarthritis and cartilage, 2020. **28**(7): p. 932-940.

10. Pareek N, K.P., et al., *A practical risk score for early prediction of neurological outcome after out-of-hospital cardiac arrest: MIRACLE2.* European heart journal, 2020.

11. Zelis N, B.J., et al., *A new simplified model for predicting 30-day mortality in older medical emergency department patients: The rise up score.* European journal of internal medicine, 2020. **77**: p. 36-43.

12. Roshanov PS, G.G., et al., *Preoperative prediction of Bleeding Independently associated with Mortality after noncardiac Surgery (BIMS): an international prospective cohort study.* British journal of anaesthesia, 2020.

13. Sutradhar R, B.L., *Comparing an Artificial Neural Network to Logistic Regression for Predicting ED Visit Risk Among Patients With Cancer: A Population-Based Cohort Study.* Journal of pain and symptom management, 2020. **60**(1): p. 1-9.

14. Kiddle SJ, W.H., S. SR, and Q. JK, *Prediction of five-year mortality after COPD diagnosis using primary care records.* PloS one, 2020. **15**(7): p. e0236011.

15. Willems SJ, C.M., et al., *Baseline Patient Characteristics Commonly Captured Before Surgery Do Not Accurately Predict Long-Term Outcomes of Lumbar Microdiscectomy Followed by Physiotherapy.* Spine, 2020. **45**(14): p. E885-E891.

16. Nguyen TL, L.S., et al., *Interval breast cancer risk associations with breast density, family history and breast tissue aging.* International journal of cancer, 2020. **147**(2): p. 375-382.

17. Bai W, C.J., et al., *A Predictive Model for the Identification of Cardiac Effusions Misclassified by Light's Criteria.* Laboratory medicine, 2020. **51**(4): p. 370-376.

18. Raita Y, C.C.J., et al., *Machine learning-based prediction of acute severity in infants hospitalized for bronchiolitis: a multicenter prospective study.* Scientific reports, 2020. **10**(1): p. 10979.

19. Abujaber A, F.A., Gammoh D, et al., *Using trauma registry data to predict prolonged mechanical ventilation in patients with traumatic brain injury: Machine learning approach.* PloS one, 2020. **15**(7): p. e0235231.

20. Knoepfel A, P.R., Lefering R and P. HC, *The AdHOC (age, head injury, oxygenation, circulation) score: a simple assessment tool for early assessment of severely injured patients with major fractures.* European journal of trauma and emergency surgery : official publication of the European Trauma Society, 2020.

21. Merhe A, L.M., Hout M, et al., *Development of a novel nomogram incorporating platelet-to-lymphocyte ratio for the prediction of lymph node involvement in prostate carcinoma.* Urologic oncology, 2020.

22. Roposch A, P.E., et al., *Predicting developmental dysplasia of the hip in at-risk newborns.* BMC musculoskeletal disorders, 2020. **21**(1): p. 442.

23. Togawa A, Y.M., et al., *Development of risk factor-based scoring system for detection of hypervirulent Klebsiella pneumoniae bloodstream infections.* Gut pathogens, 2020. **12**: p. 34.

24. Zeadna A, K.N., et al., *Prediction of sperm extraction in non-obstructive azoospermia patients: a machine-learning perspective.* Human reproduction (Oxford, England), 2020. **35**(7): p. 1505-1514.

25. Anderson AB, G.C., Balazs GC, et al., *Can Predictive Modeling Tools Identify Patients at High Risk of Prolonged Opioid Use After ACL Reconstruction?* Clinical orthopaedics and related research, 2020. **478**(7): p. 0-1618.

26. Kao AM, M.S., et al., *The CELIOtomy Risk Score: An effort to minimize futile surgery with analysis of early postoperative mortality after emergency laparotomy.* Surgery, 2020.

27. Chen B, Z.L., Wang D, et al., *Nomogram to predict postpartum hemorrhage in cesarean delivery for women with scarred uterus: A retrospective cohort study in China.* The journal of obstetrics and gynaecology research, 2020.

28. Duceau B, A.J., Bellenfant F, et al., *Prehospital triage of acute aortic syndrome using a machine learning algorithm.* The British journal of surgery, 2020. **107**(8): p. 995-1003.

29. Yang B, S.K., et al., *Large population-based study using the SEER database: is endoscopic resection appropriate for early gastric cancer patients in the United States?* Scandinavian journal of gastroenterology, 2020: p. 1-9.

30. Zhao B, H.B., et al., *Predictive Model for Pulmonary Embolism in Patients with Deep Vein Thrombosis.* Annals of vascular surgery, 2020. **66**: p. 334-343.

31. Bender C, C.S., Pape-Haugaard L, et al., *Assessment of Simple Bedside Wound Characteristics for a Prediction Model for Diabetic Foot Ulcer Outcomes.* Journal of diabetes science and technology, 2020: p. 1932296820942307.

32. Chattot C, D.X., et al., *Preoperative predictors and a prediction score for perception of improvement after mesh prolapse surgery.* International urogynecology journal, 2020. **31**(7): p. 1393-1400.

33. Han C, M.W., An J, J. L, and C. P, *Early morning off in patients with Parkinson's disease: a Chinese nationwide study and a 7-question screening scale.* Translational neurodegeneration, 2020. **9**(1): p. 29.

34. Cotton CC, B.R., et al., *A Model Using Clinical and Endoscopic Characteristics Identifies Patients at Risk for Eosinophilic Esophagitis According to Updated Diagnostic Guidelines.* Clinical gastroenterology and hepatology : the official clinical practice journal of the American Gastroenterological Association, 2020.

35. Monamele CG, K.-N.C., et al., *Clinical signs predictive of influenza virus infection in Cameroon.* PloS one, 2020. **15**(7): p. e0236267.

36. Chen CY, L.C., et al., *Check point to get adequate weight loss within 6-months after laparoscopic sleeve gastrectomy for morbid obesity in Asian population.* Scientific reports, 2020. **10**(1): p. 12788.

37. Tsilimigras DI, S.K., et al., *Very Early Recurrence After Liver Resection for Intrahepatic Cholangiocarcinoma: Considering Alternative Treatment Approaches.* JAMA surgery, 2020.

38. Jiang E, G.H., et al., *Predicting and comparing postoperative infections in different stratification following PCNL based on nomograms.* Scientific reports, 2020. **10**(1): p. 11337.

39. Vesale E, R.H., et al., *Predictive approach in managing voiding dysfunction after surgery for deep endometriosis: a personalized nomogram.* International urogynecology journal, 2020.

40. Capretti G, C.M., et al., *Enhanced Recovery After Pancreatic Surgery Does One Size Really Fit All? A Clinical Score to Predict the Failure of an Enhanced Recovery Protocol After Pancreaticoduodenectomy.* World journal of surgery, 2020.

41. Gök G, K.M., S. ÜY, and Z. M, *A New Risk Score to Predict In-Hospital Mortality in Elderly Patients With Acute Heart Failure: On Behalf of the Journey HF-TR Study Investigators.* Angiology, 2020: p. 3319720941758.

42. Wong GJY, P.J., L. YY, and L. CCH, *Refeeding Hypophosphatemia in Patients Receiving Parenteral Nutrition: Prevalence, Risk Factors, and Predicting Its Occurrence.* Nutrition in clinical practice : official publication of the American Society for Parenteral and Enteral Nutrition, 2020.

43. Lian H, X.X., et al., *Early prediction of cerebral-cardiac syndrome after ischemic stroke: the PANSCAN scale.* BMC neurology, 2020. **20**(1): p. 272.

44. Muttai H, G.B., et al., *Development and Validation of a Sociodemographic and Behavioral Characteristics-Based Risk-Score Algorithm for Targeting HIV Testing Among Adults in Kenya.* AIDS and behavior, 2020.

45. Foroushani HM, H.A., et al., *Quantitative Serial CT Imaging-Derived Features Improve Prediction of Malignant Cerebral Edema after Ischemic Stroke.* Neurocritical care, 2020.

46. Kwak HS, P.J., *Can Computed Tomographic Angiography Be Used to Predict Who Will Not Benefit from Endovascular Treatment in Patients with Acute Ischemic Stroke? The CTA-ABC Score.* Journal of Korean Neurosurgical Society, 2020. **63**(4): p. 470-476.

47. Gagnon I, T.E., et al., *Parent-Child Agreement on Postconcussion Symptoms in the Acute Postinjury Period.* Pediatrics, 2020. **146**(1).

48. Niyongombwa I, S.I., et al., *Kigali Surgical Sepsis (KiSS) Score: A New Tool to Predict Outcomes in Surgical Patients with Sepsis in Low- and Middle-Income Settings.* World journal of surgery, 2020.

49. Tseng IC, C.I., et al., *Predictors of Acute Mortality After Open Pelvic Fracture: Experience From 37 Patients From A Level I Trauma Center.* World journal of surgery, 2020.

50. Ellis-Kahana J, S.A., et al., *Developing a model for predicting venous thromboembolism in obese pregnant women in a national study.* Thrombosis research, 2020. **191**: p. 42-49.

51. Feghali J, M.E., et al., *Novel Risk Calculator for Suboccipital Decompression for Adult Chiari Malformation.* World neurosurgery, 2020. **139**: p. 526-534.

52. Ma J, S.X., et al., *Development and validation of a risk stratification model for screening suspected cases of COVID-19 in China.* Aging, 2020. **12**.

53. Vivier-Chicoteau J, L.J., et al., *Development and internal validation of a diagnostic score for gastric linitis plastica.* Gastric cancer : official journal of the International Gastric Cancer Association and the Japanese Gastric Cancer Association, 2020. **23**(4): p. 639-647.

54. Xiao J, X.Q., et al., *Discriminating Malignancy in Thyroid Nodules: The Nomogram Versus the Kwak and ACR TI-RADS.* Otolaryngology--head and neck surgery : official journal of American Academy of Otolaryngology-Head and Neck Surgery, 2020: p. 194599820939071.

55. Sim JA, K.Y., et al., *The major effects of health-related quality of life on 5-year survival prediction among lung cancer survivors: applications of machine learning.* Scientific reports, 2020. **10**(1): p. 10693.

56. Mejia-Otero JD, A.S. and W. PC, *Risk factors for hospitalization in youth with type 1 diabetes: Development and validation of a multivariable prediction model.* Pediatric diabetes, 2020.

57. Delparte JJ, F.H., S. CY, and B. AS, *Development of the spinal cord injury pressure sore onset risk screening (SCI-PreSORS) instrument: a pressure injury risk decision tree for spinal cord injury rehabilitation.* Spinal cord, 2020.

58. Shoenbill K, S.Y., et al., *Identifying patterns and predictors of lifestyle modification in electronic health record documentation using statistical and machine learning methods.* Preventive medicine, 2020. **136**: p. 106061.

59. Uchida K, Y.S., et al., *Simplified Prehospital Prediction Rule to Estimate the Likelihood of 4 Types of Stroke: The 7-Item Japan Urgent Stroke Triage (JUST-7) Score.* Prehospital emergency care : official journal of the National Association of EMS Physicians and the National Association of State EMS Directors, 2020: p. 1-14.

60. Rothenberg KA, G.E., et al., *Assessment of the Risk Analysis Index for Prediction of Mortality, Major Complications, and Length of Stay in Patients who Underwent Vascular Surgery.* Annals of vascular surgery, 2020. **66**: p. 442-453.

61. Benoit L, B.V., et al., *Nomogram Predicting the Likelihood of Parametrial Involvement in Early-Stage Cervical Cancer: Avoiding Unjustified Radical Hysterectomies.* Journal of clinical medicine, 2020. **9**(7).

62. Qin L, Y.Y., et al., *A predictive model and scoring system combining clinical and CT characteristics for the diagnosis of COVID-19.* European radiology, 2020: p. 1-11.

63. Wu L, C.W., S. Y, and W. L, *Predicting treatment failure risk in a Chinese Drug-Resistant Tuberculosis with surgical therapy: Development and assessment of a new predictive nomogram.* International journal of infectious diseases : IJID : official publication of the International Society for Infectious Diseases, 2020. **96**: p. 88-93.

64. Zhan L, W.X. and Z. LX, *Nomogram Model for Predicting Risk of Postoperative Delirium After Deep Brain Stimulation Surgery in Patients Older Than 50 Years with Parkinson Disease.* World neurosurgery, 2020. **139**: p. e127-e135.

65. Rocio LG, A.U., et al., *Interleukin-6-based mortality risk model for hospitalised COVID-19 patients.* The Journal of allergy and clinical immunology, 2020.

66. Tennenhouse LG, M.R., B. CN, and L. LM, *Machine-learning models for depression and anxiety in individuals with immune-mediated inflammatory disease.* Journal of psychosomatic research, 2020. **134**: p. 110126.

67. Vitzthum LK, R.P., et al., *Predicting Persistent Opioid Use, Abuse, and Toxicity Among Cancer Survivors.* Journal of the National Cancer Institute, 2020. **112**(7): p. 720-727.

68. Xiao LS, Z.W., et al., *Development and validation of the HNC-LL score for predicting the severity of coronavirus disease 2019.* EBioMedicine, 2020. **57**: p. 102880.

69. Ho-Pham LT, D.M., V. LH, and N. TV, *Development of a model for identification of individuals with high risk of osteoporosis.* Archives of osteoporosis, 2020. **15**(1): p. 111.

70. Yin LX, S.A., et al., *An internally validated diagnostic tool for acute invasive fungal sinusitis.* International forum of allergy & rhinology, 2020.

71. Baimas-George M, W.M., et al., *A pre-operative platelet transfusion algorithm for patients with cirrhosis and hepatocellular carcinoma undergoing laparoscopic microwave ablation.* Surgical endoscopy, 2020.

72. Iriondo M, T.M., et al., *Prediction of mortality in very low birth weight neonates in Spain.* PloS one, 2020. **15**(7): p. e0235794.

73. Jacob M, R.J., et al., *Predicting lung nodules malignancy.* Pulmonology, 2020.

74. Ladios-Martin M, F.-d.-M.J., et al., *Predictive Modeling of Pressure Injury Risk in Patients Admitted to an Intensive Care Unit.* American journal of critical care : an official publication, American Association of Critical-Care Nurses, 2020. **29**(4): p. e70-e80.

75. Raseta M, B.A., et al., *A novel toolkit for the prediction of clinical outcomes following mechanical thrombectomy.* Clinical radiology, 2020.

76. Tago M, K.N., et al., *New predictive models for falls among inpatients using public ADL scale in Japan: A retrospective observational study of 7,858 patients in acute care setting.* PloS one, 2020. **15**(7): p. e0236130.

77. Xue M, S.Y., et al., *A nomogram model for screening the risk of diabetes in a large-scale Chinese population: an observational study from 345,718 participants.* Scientific reports, 2020. **10**(1): p. 11600.

78. Yamamoto M, O.T., et al., *Clinical risk model for predicting 1-year mortality after transcatheter aortic valve replacement.* Catheterization and cardiovascular interventions : official journal of the Society for Cardiac Angiography & Interventions, 2020.

79. Zhang M, L.D., et al., *Gastrointestinal bleeding in patients admitted to cardiology: risk factors and a new risk score.* Hellenic journal of cardiology : HJC = Hellenike kardiologike epitheorese, 2020.

80. Naim MY, P.M., et al., *Development and Validation of a Seizure Prediction Model in Neonates Following Cardiac Surgery.* The Annals of thoracic surgery, 2020.

81. Balachandren N, S.M., et al., *Ovarian reserve as a predictor of cumulative live birth.* European journal of obstetrics, gynecology, and reproductive biology, 2020. **252**: p. 273-277.

82. Hou N, X.J., et al., *Development and Validation of a Nomogram for Individually Predicting Pathologic Complete Remission After Preoperative Chemotherapy in Chinese Breast Cancer: A Population-Based Study.* Clinical breast cancer, 2020.

83. Zhang P, B.Y., T. Z, and W. F, *Use of Nutrition Risk in Critically ill (NUTRIC) scoring system for nutritional risk assessment and prognosis prediction in critically ill neurological patients: a prospective observational study.* JPEN. Journal of parenteral and enteral nutrition, 2020.

84. Zhang P, L.Y., et al., *Risk factors analysis and a nomogram model establishment for late postoperative seizures in patients with meningioma.* Journal of clinical neuroscience : official journal of the Neurosurgical Society of Australasia, 2020.

85. Tseng PY, C.Y., et al., *Prediction of the development of acute kidney injury following cardiac surgery by machine learning.* Critical care (London, England), 2020. **24**(1): p. 478.

86. Wu Q, L.J., et al., *Efficacy of interleukin-6 in combination with D-dimer in predicting early poor postoperative prognosis after acute stanford type a aortic dissection.* Journal of cardiothoracic surgery, 2020. **15**(1): p. 172.

87. Chu R, C.W., et al., *Predicting the Risk of Adverse Events in Pregnant Women With Congenital Heart Disease.* Journal of the American Heart Association, 2020. **9**(14): p. e016371.

88. Yamamoto R, S.T., A. N, and S. J, *Modified abbreviated burn severity index as a predictor of in-hospital mortality in patients with inhalation injury: development and validation using independent cohorts.* Surgery today, 2020.

89. Benirschke RC, G.T., *Detection of Falsely Elevated Point-of-Care Potassium Results Due to Hemolysis Using Predictive Analytics.* American journal of clinical pathology, 2020. **154**(2): p. 242-247.

90. Luzzago S, d.C.O., et al., *A novel nomogram to identify candidates for active surveillance amongst patients with International Society of Urological Pathology (ISUP) Grade Group (GG) 1 or ISUP GG2 prostate cancer, according to multiparametric magnetic resonance imaging findings.* BJU international, 2020. **126**(1): p. 104-113.

91. Wang S, T.S., et al., *Development and validation of a novel scoring system developed from a nomogram to identify malignant pleural effusion.* EBioMedicine, 2020. **58**: p. 102924.

92. Klim SM, A.F., et al., *Combined serum biomarker analysis shows no benefit in the diagnosis of periprosthetic joint infection.* International orthopaedics, 2020.

93. Kojima T, N.T., et al., *Keratoconus Screening Using Values Derived From Auto-Keratometer Measurements: A Multicenter Study.* American journal of ophthalmology, 2020. **215**: p. 127-134.

94. Vieceli T, F.C., et al., *A predictive score for COVID-19 diagnosis using clinical, laboratory and chest image data.* The Brazilian journal of infectious diseases : an official publication of the Brazilian Society of Infectious Diseases, 2020.

95. Kalhan TA, U.L.C., et al., *Caries Risk Prediction Models in a Medical Health Care Setting.* Journal of dental research, 2020. **99**(7): p. 787-796.

96. Puar TH, L.W., et al., *Aldosterone-potassium ratio predicts primary aldosteronism subtype.* Journal of hypertension, 2020. **38**(7): p. 1375-1383.

97. Kuo TJ, H.C., et al., *Nomogram for pneumonia prediction among children and young people with cerebral palsy: A population-based cohort study.* PloS one, 2020. **15**(7): p. e0235069.

98. Sun W, X.L., et al., *Development and validation of two aspiration prediction models in patients receiving nasogastric feeding.* Journal of nursing management, 2020.

99. W, W., Y. Z, and O. Q, *A nomogram to predict skip metastasis in papillary thyroid cancer.* World journal of surgical oncology, 2020. **18**(1): p. 167.

100. Chung WC, S.C., et al., *Novel mechanical ventilator weaning predictive model.* The Kaohsiung journal of medical sciences, 2020.

101. Zheng WX, H.G., et al., *Establishment and internal validation of preoperative nomograms for predicting the possibility of testicular salvage in patients with testicular torsion.* Asian journal of andrology, 2020.

102. Du X, M.J., et al., *Predicting in-hospital mortality of patients with febrile neutropenia using machine learning models.* International journal of medical informatics, 2020. **139**: p. 104140.

103. Li X, F.X., et al., *A Novel Risk Stratification Score for Sudden Cardiac Death Prediction in Middle-Aged, Nonischemic Dilated Cardiomyopathy Patients: The ESTIMATED Score.* The Canadian journal of cardiology, 2020. **36**(7): p. 1121-1129.

104. Pan X, X.X., et al., *Risk Prediction for Non-alcoholic Fatty Liver Disease Based on Biochemical and Dietary Variables in a Chinese Han Population.* Frontiers in public health, 2020. **8**: p. 220.

105. Tang X, J.W., et al., *Predicting poor response to neoadjuvant chemoradiotherapy for locally advanced rectal cancer: Model constructed using pre-treatment MRI features of structured report template.* Radiotherapy and oncology : journal of the European Society for Therapeutic Radiology and Oncology, 2020. **148**: p. 97-106.

106. Chen Y, G.J., et al., *A Novel Prediction Model for Significant Liver Fibrosis in Patients with Chronic Hepatitis B.* BioMed research international, 2020. **2020**: p. 6839137.

107. Fan Y, C.M. and X. L, *Distinction and Potential Prediction of Lung Metastasis in Patients with Malignant Primary Osseous Spinal Neoplasms.* Spine, 2020. **45**(13): p. 921-929.

108. Gu Y, W.Q., et al., *Is Cervical Traction Effective in Chronic Nonspecific Neck Pain Patients With Unsatisfactory NSAID Control? A Nomogram to Predict Effectiveness.* World neurosurgery, 2020. **139**: p. e245-e254.

109. Shimizu Y, H.S., et al., *New Model for Predicting Malignancy in Patients With Intraductal Papillary Mucinous Neoplasm.* Annals of surgery, 2020. **272**(1): p. 155-162.

110. Sun Y, K.V., et al., *Epidemiological and Clinical Predictors of COVID-19.* Clinical infectious diseases : an official publication of the Infectious Diseases Society of America, 2020. **71**(15): p. 786-792.

111. Zhao Y, F.L., C. L, and B. S, *Application of data mining for predicting hemodynamics instability during pheochromocytoma surgery.* BMC medical informatics and decision making, 2020. **20**(1): p. 165.

112. Zhang Y, S.R., et al., *Establishment of a Risk Prediction Model for Non-alcoholic Fatty Liver Disease in Type 2 Diabetes.* Diabetes therapy : research, treatment and education of diabetes and related disorders, 2020.

113. Cai YL, L.Y., et al., *A Novel Nomogram Predicting Distant Metastasis in T1 and T2 Gallbladder Cancer: A SEER-based Study.* International journal of medical sciences, 2020. **17**(12): p. 1704-1712.

114. Zhang YZ, Z.L., et al., *A mid-pregnancy risk prediction model for gestational diabetes mellitus based on the maternal status in combination with ultrasound and serological findings.* Experimental and therapeutic medicine, 2020. **20**(1): p. 293-300.

115. Du Z, Y.Y., et al., *Accurate Prediction of Coronary Heart Disease for Patients With Hypertension From Electronic Health Records With Big Data and Machine-Learning Methods: Model Development and Performance Evaluation.* JMIR medical informatics, 2020. **8**(7): p. e17257.

116. Wang Z, X.J., et al., *Nomogram Analysis and Internal Validation to Predict the Risk of Cystobiliary Communication in Patients Undergoing Hydatid Liver Cyst Surgery.* World journal of surgery, 2020.

117. Zhao Z, C.A., et al., *Prediction model and risk scores of ICU admission and mortality in COVID-19.* PloS one, 2020. **15**(7): p. e0236618.

118. Lv ZH, Y.N., *Construction of a Risk Prediction Model for Fever After Painless Bronchoscopy.* Medical science monitor : international medical journal of experimental and clinical research, 2020. **26**: p. e924911.
